# Spatial and Temporal Distribution of Information Processing in the Human Dorsal Anterior Cingulate Cortex

**DOI:** 10.3389/fnhum.2022.780047

**Published:** 2022-03-18

**Authors:** Conor Keogh, Alceste Deli, Amir Puyan Divanbeighi Zand, Mark Jernej Zorman, Sandra G. Boccard-Binet, Matthew Parrott, Charalampos Sigalas, Alexander R. Weiss, John Frederick Stein, James J. FitzGerald, Tipu Z. Aziz, Alexander L. Green, Martin John Gillies

**Affiliations:** ^1^Nuffield Department of Surgical Sciences, University of Oxford, Oxford, United Kingdom; ^2^St Hilda’s College, University of Oxford, Oxford, United Kingdom; ^3^Department of Pharmacology, University of Oxford, Oxford, United Kingdom; ^4^Department of Neurology, Johns Hopkins University, Baltimore, MD, United States; ^5^Department of Physiology, Anatomy and Genetics, University of Oxford, Oxford, United Kingdom

**Keywords:** human cortex, electrophysiology, decision making, information theory, error

## Abstract

The dorsal anterior cingulate cortex (dACC) is a key node in the human salience network. It has been ascribed motor, pain-processing and affective functions. However, the dynamics of information flow in this complex region and how it responds to inputs remain unclear and are difficult to study using non-invasive electrophysiology. The area is targeted by neurosurgery to treat neuropathic pain. During deep brain stimulation surgery, we recorded local field potentials from this region in humans during a decision-making task requiring motor output. We investigated the spatial and temporal distribution of information flow within the dACC. We demonstrate the existence of a distributed network within the anterior cingulate cortex where discrete nodes demonstrate directed communication following inputs. We show that this network anticipates and responds to the valence of feedback to actions. We further show that these network dynamics adapt following learning. Our results provide evidence for the integration of learning and the response to feedback in a key cognitive region.

## Introduction

The dorsal anterior cingulate cortex (dACC) is located in the medial prefrontal cortex. It is a key node in the human salience network (Seeley et al., 2007; Uddin, 2015). A variety of specific functions have been ascribed to the dACC based on human stimulation studies. These include internally generated movement, affective and pain-processing (Misra and Coombes, 2015; Oane et al., 2020), error detection (Holroyd and Coles, 2002; Hyman et al., 2013) and reward-based decision-making (Walton et al., 2003; Behrens et al., 2007). The dACC demonstrates important motor functions, distinguishing it from the more ventral ACC (Hoffstaedter et al., 2014). This region is sometimes referred to as the anterior midcingulate cortex. In this work, we follow the convention of the neurosurgical pain literature in using the dACC (Boccard et al., 2015).

Our group has previously reported on the laterality of cognitive function in the human dACC during decision making requiring a motor output (Weiss et al., 2018). The location of the dACC makes lateralization of dACC activity difficult to study in humans as it is not accessible to standard EEG recordings and the timescale of activity is too short to investigate using fMRI. Using local field potentials recorded using electrodes implanted in the human dACC, we demonstrated that the left (dominant) dACC responded to outcome valence following voluntary movement (i.e., whether feedback was positive or negative) whereas the right dACC (non-dominant) was predominantly active around the time of stimulus presentation, which we proposed to represent prediction formation.

Despite this insight into the laterality of function in the dACC, the dynamics of information processing in this important cognitive region remain unclear. Functional MRI evidence suggests the existence of asymmetries in the human salience network (Cauda et al., 2011; Zhang et al., 2019). This asymmetrical information flow appears to be contingent on outcome valence signaled by the left dACC and to influence prediction formation dynamics in the right dACC (Seo and Lee, 2007; Lütcke and Frahm, 2008; Hyman et al., 2017; Weiss et al., 2018). This is in line with action-outcome predictor models of ACC function (Alexander and Brown, 2011). Further, information transfer between hemispheres has been proposed to be important for adjusting behavioral strategies to optimize action-outcome in uncertain environments (Kennerley et al., 2006; Womelsdorf and Everling, 2015).

We hypothesized that the dACC contains a distributed network of regions that are responsible for information processing and integration in this motor and cognitive region. We further hypothesized that these discrete nodes communicate following inputs to the dACC to facilitate response integration and that these network dynamics would respond to learning strategies as the dACC adapted to task requirements.

Using electrodes implanted in the dACC bilaterally, we recorded local field potentials while human participants performed a cognitive task. We demonstrate that discrete regions respond to motor-related activity, anticipation, outcome valence and prediction error. We show that these regions communicate with fixed dynamics following inputs and that these dynamics vary as a function of task variables, providing evidence for a distributed network that processes and integrates cognitive and motor information. Further, we demonstrate that the dynamics of this network adapt over time as learning occurs, suggesting the existence of a distributed network in the dACC that dynamically responds to inputs and adapts its response.

## Materials and Methods

The collection of the dataset analyzed here was described in detail in Weiss et al. (2018). This study was carried out in accordance with the recommendations and approval of Oxfordshire Research Ethics Committee A (Ref 11/SC/0229). All subjects gave written informed consent in accordance with the Declaration of Helsinki.

### Electrode Positioning

Three right-handed subjects with medically and surgically refractory neuropathic pain (two male, one female; 42 ± 4.9 years old) underwent bilateral electrode implantation in the dACC based on MRI targets. Electrode tips were targeted 20 mm posterior to the frontal horn and 8 mm lateral to the midline. Diffusion tensor imaging and tractography confirmed connectivity predominantly to the supplementary motor area bilaterally in all three subjects.

Each lead contained four 1.5 mm circumferential electrodes, each separated by 1.5 mm. Bipolar recordings were made from adjacent electrode pairs. Supplementary Figure 1 indicates electrode positions in subject native space. Data were high-pass filtered at 0.5 Hz and digitized at 2,048 Hz. Data were notch filtered at 50, 100, and 150 Hz using zero-phase shift Bessel filters.

### Behavioral Task

The subjects had performed an Intra-Extra Dimensional Set Shifting task, a variation of the Wisconsin Card Sorting Test. This is described in detail in Weiss et al. (2018). It is a test of rule acquisition and reversal. It tests visual discrimination, attentional set formation maintenance, set shifting and flexibility of attention (Cambridge Cognition, Cambridge, United Kingdom).

Briefly, subjects must learn nine rules governing correct object selection that vary during the task from a choice of two abstract solid filled objects or object pairs (solid filled object plus white line object). These vary in their spatial and morphological arrangements. Blank squares appear on screen at the start of a trial. This is followed by object presentation. Participants must then make a selection based on the current rule. Object selection elicits audio-visual feedback which indicates if the choice was correct or incorrect.

Once a rule is learned, evidenced by 6 correct object choices in a row, the rule changes without any cue to the participant. The subject passes the task if they successfully learn all nine rules. If the subject fails to learn an individual rule, evidenced by failure to attain six correct in a row within 50 attempts, the task is failed. Supplementary Figure 2 demonstrates the task workflow and how fixed timepoints in the task can be time-locked to the electrophysiological data.

### Data Pre-processing

Recordings were divided into trials. Feedback and stimulus presentation epochs were extracted for each trial.

Two second epochs were extracted, time-locked to the event (feedback or stimulus presentation). One second of data before and one second after the event were included in each epoch. All individual epochs were then baseline corrected and normalized to unit variance in order to standardize across individuals and across sessions. This was performed by subtracting the trial mean from each timepoint and dividing by the trial standard deviation, i.e., $\hat{x_{t}}=\frac{x_{t}-\mu }{\sigma }\,\forall t\in T$.

All feedback event-related potential epochs were labeled depending on the valence of the feedback (correct or incorrect). Stimulus presentation epochs were labeled according to the feedback valence of the preceding trial, whether the rule was learned or unlearned, and whether the trial occurred early or late in the current rule sequence in order to investigate the factors influencing the response to stimulus presentation as outlined below.

All data was manually inspected to ensure appropriate recording quality and epochs with significant artifact were manually rejected.

A semi-automated artifact rejection method was then used, based on a statistical time-domain threshold. Of 4842 recordings (six electrodes in 807 trials), 131 feedback epochs and 148 stimulus presentation epochs were rejected. Discarded epochs were replaced with the mean of remaining recordings on that electrode in order to maintain a balanced number of trials between conditions for within-subjects comparisons.

### Waveform Analysis

The electrophysiological response to events was characterized by determining the intervals in which the time-domain signal was statistically significant across trials or between conditions, based on a significance threshold of *p* < 0.0001, Bonferroni corrected for the number of samples tested (e.g., for analysis of a full two-second epoch recorded at 2048 Hz, a threshold of 0.0001 ÷ 4097 = 2 × 10^–8^ was used. This represents a *p* < 0.01% chance of a type I error, corrected for the number of comparisons made).

### Source Decomposition

Electrode recordings were decomposed into statistically independent sources of activity using independent component analysis (Hyvärinen and Oja, 2000). This allows us to separate the signals recorded on each electrode, which represent a mixture of local neuronal subpopulations with varying contributions, into individual signals which best represent independent activities, possibly representing the underlying neuronal populations. This allows us to investigate the behavior of neural subpopulations within the dACC in much greater detail and to characterize the network producing event-related activity.

### Network Communication

Communication between components of the dACC network was investigated. Coherence is formally defined as:


$$C_{x\,y}\,\left(f\right)=\frac{{\left|G_{x\,y}\,\left(f\right)\right|}^{2}}{G_{x\,x}\,\left(f\right)\,G_{y\,y}\,\left(f\right)}$$

where *G*_*xy*_(*f*) is the cross-spectral density between x and y and *G*_*xx*_(*f*) and

*G*_*yy*_(*f*) represent the autospectral densities of x and y.

A directional coherence estimate was calculated between signals, over a range of time lags to investigate the directionality and temporal dynamics of communication within the dACC network – i.e., for assessing network communication during a range spanning {t_0_ … t_*n*_}, the second signal was tested over {t_0_ + τ… t_*n*_ + τ} for a range of τ in order to identify the time range in which communication between populations was greatest. This was repeated in both directions in order to assess directionality of communication.

The overall pattern of network communication within the network was compared between conditions by performing principal component analysis on the pairwise coherence measures. By identifying the component that accounts for most of the variance in the network communication data within the high dimensional space of pairwise connectivity measures, we can compare the overall network pattern directly while maintaining the structure of the underlying data (Keogh et al., 2019). The pairwise measures of connectivity define a high-dimensional space; the high covariance between pairwise measure indicates that network activity in this space is structured. By taking advantage of this structure using principal component analysis, we can compare the effect of task variables on the overall pattern of network communication, rather than as a large number of individual pairwise comparisons. This provides a more global picture of the impact of task events on network dynamics. The weights of the first three principal components, comprising most of the variability in network activity, over the pairwise connectivity metrics can be visualized in Supplementary Figure 3.

To investigate the effects of learning on network dynamics, we calculated the non-directional coherence between signals for low (30–60 Hz) and high (60–100 Hz) gamma, beta (12.5–30 Hz) and theta (4–8 Hz) frequency bands. This provides greater insight into the nature of the changes in network dynamics induced by learning.

The dynamics of directional communication within the network were further characterized using Granger causality (Granger, 1988) of the source-space representation. This allows us to test whether knowledge of the past values of one of our signals increases our ability to predict future values of another signal; if this is the case, we infer that there is directional transfer of information between neuronal populations within the network. Formally, this is tested by determining whether the predictions given by the autoregression of y:


$$y_{t}=a_{0}+a_{1}\,y_{t-1}+\ldots +a_{n}\,y_{t-n}+\varepsilon_{t}$$

are improved by inclusion of lagged values of x:


$$y_{t}=a_{0}+a_{1}\,y_{t-1}+\ldots +a_{n}\,y_{t-n}+b_{p}\,x_{t-p}+\ldots \,b_{q}\,x_{t-q}+\varepsilon_{t}$$

Due to the event-related nature of the feedback-locked signal, the signals were first-differenced (i.e., $y_{t}^{\prime }=y_{t}-y_{t-1}$) prior to analysis to improve stationarity. This was repeated over a range of lags in both directions in order to characterize the direction and temporal dynamics of communication within the dACC network.

Analysis was performed using signals isolated within specific windows of interest (–200 to 0 ms and 100 to 500 ms) and lags from 0 to 50 ms were included. This allowed for analysis of directed communication in the windows of interest while avoiding the potential confounding effects of including lagged data from previous trials that may have artificially increased the apparent predictability of responses.

A parametric estimate of Granger is often difficult to calculate due to overfitting. We therefore assessed if our results were consistent for a variety of model orders over multiple iterations (deployed to determine the best data fit). Since the values of the parametric and non-parametric model order are inversely related to prediction error, we ensured that values were positive for all acceptable iterations (model order 20, 30, 40, and 50).

We supported our Granger causality analysis by examining information dynamics within the dACC network using a multivariate implementation of transfer entropy (Novelli et al., 2019). This quantifies the directional information flow within the network by examining the reduction in uncertainty of the future values of one source’s activity using previous values of all other sources. Formally, this assesses the reduction in entropy, a measure of uncertainty:


$$H\,\left(X\right)=\sum_{i=1}^{n}P\,\left(x_{i}\right)\,\log_{2}P\,\left(x_{i}\right)$$

where *P*(*x*_*i*_) is the probability of *x*_*i*_, when we have knowledge of the past information of another signal:


$$T_{X\to Y}=H\,\left(Y_{t}|Y_{t-1:t-L}\right)-H\,\left(Y_{t}|Y_{t-1:t-L},X_{t-1:t-L}\right)$$

This approach offers a number of advantages: particularly, it is a multivariate metric, allowing us to make use of the structure of our data, and it is model free, i.e., it does not require us to assume that the underlying model is linear, overcoming some of the limitations of Granger causality (noting also that transfer entropy reduces to Granger causality in the case of auto-regressive signals). Analyzing the multivariate transfer entropy of the independent sources of our data allows us to take advantage of the structure of our data to quantify the dynamics of information flow between neuronal populations in the dACC.

### Statistical Analysis

All statistical significance tests of feedback epochs were performed with a strict significance threshold of *p* < 0.0001. Where multiple comparisons were made, significance thresholds were Bonferroni corrected, i.e., the threshold for a statistically significant result was adjusted to *p* < 0:0001/n, where n is the number of comparisons made. Analyses were restricted to the broadband signal unless otherwise stated. Where data was high-dimensional with a high degree of covariance, as in the pairwise coherence measures, pairwise statistical tests were avoided; dimensionality was reduced using principal component analysis and the components that accounted for the greatest degree of variance compared.

For statistical comparisons of connectivity differences between rule learned and unlearned epochs at stimulus presentation, we used a two-sample t-test to compare values between areas and trial groups (statistical significance at 0.05 level). All *p*-values of these analyses were then corrected for their respective number of multiple comparisons by Bonferroni correction. This method was deemed appropriate since the type 1 error rate was controlled at 5% given all groups’ sample size.

All analyses were carried out in MATLAB and Python using bespoke in-house scripts, incorporating the FieldTrip (Oostenveld et al., 2011) and SciPy signal analysis toolboxes.

## Results

### The Dorsal Anterior Cingulate Cortex Network Anticipates and Responds to Feedback

**FIGURE 1:**
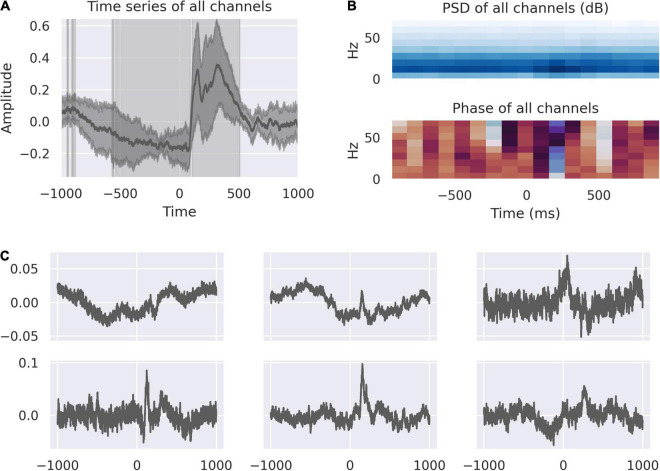
The dACC responds to feedback. **(A)** The average feedback-locked response across all channels; shading represents the variance between electrodes. The highlighted regions demonstrate where the response is statistically significant at *p* < 0.0001, Bonferroni corrected. This identifies a movement correlated and anticipatory window beginning at 528 ms before feedback and a response window from +100 ms to +500 ms post-feedback. **(B)** Power spectral density and phase spectrum across all channels, time-locked to feedback. The time-domain response is associated with activity in the 10–20 Hz range. The pre-feedback window is marked by a phase reset at roughly –200 ms, corresponding with a reduction in variance in the average signal. **(C)** Source decomposition of all channels, representing the activity of statistically independent populations. The activity of these populations correspond to the components of the overall signal, including pre-feedback and early and late post-feedback responses.

Averaged traces from all channels from left and right dACC showed a multi-phasic response during object selection and feedback (Figure 1A).

There is a negative-going response starting approximately 500ms before feedback. This corresponds to the period in which movement is occurring in the right hand to touch the screen. At –200 ms, there is a reduction in variance across all channels and a phase reset indicating increased coherence in the dACC network immediately prior to feedback (Figure 1B).

After feedback there is a double-peaked response, corresponding to the early and late response described in Weiss et al. (2018). We know from our previous analysis that the left dACC responds to valence of feedback, showing significantly greater activity in response to incorrect feedback. We decomposed the averaged peri-feedback signal into its component parts to study the distribution of responses to events peri-feedback. An analysis of these sources show that the first independent component represents a source with most marked activity at –200 ms. Independent components, 2 and 6, are related to the earlier portion of the feedback window with peaks of activity around –500 ms when movement is occurring. Independent components 2, 3, 4, 5, and 6 all show responses at 200 ms post-feedback, while 3, 4, and 6 all show a second peak of activity at 400 ms post-feedback (Figure 1C).

### Dorsal Anterior Cingulate Cortex Functions Are Mediated by a Distributed Network Between Right and Left Dorsal Anterior Cingulate Cortex

**FIGURE 2:**
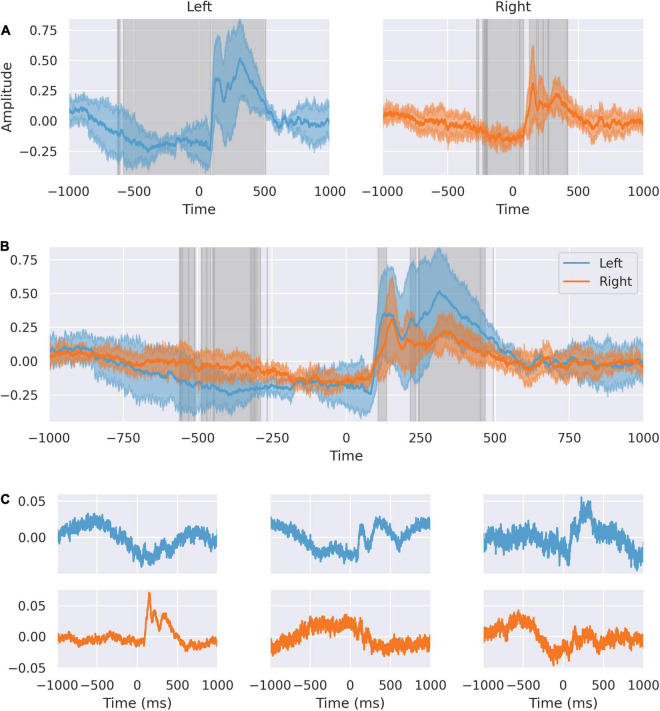
The dACC response is lateralized. **(A)** Response of left and right dACC. Highlighted regions show where activity is statistically significant. Left dACC prefeedback interval begins earlier corresponding to when movement is occurring. **(B)** Response of left and right superimposed. Highlighted regions show statistically significant differences between left and right. There is an interval from 106 to 135 ms representing the peak lag between sides. The 242–566 ms interval shows differences in the response to feedback between left and right. **(C)** Source decomposition of signals from left (blue) and right (orange) dACC. Independent sources within the left and right dACC respond differently, with a stronger feedback response in the left ACC and more predictive activity in the right ACC.

We further analyzed the spatial distribution of independent sources of activity in the dACC in the peri-feedback epoch (Figure 2). We compared the averaged responses of right and left dACC. Activity in the left significantly varies from right from approximately –500 ms before feedback to –200 ms. At –200 ms before feedback, activity synchronizes between right and left dACC. In response to feedback, we confirmed the double-peaked nature of the response on both sides.

The different responses in left and right dACC suggest a lateralization of function in the peri-feedback period. We decomposed the averaged feedback-locked signals from the left and right dACC into distinct sources to assess the relative contributions on each side to the overall dACC network response (Figure 2).

On the left side, the second source has a double-peaked structure post-feedback, and likely relates to the feedback response seen in the average signal. Similarly, the third source shows a response at +200 to +500 ms post-feedback, again suggesting that this left-sided source is related to feedback. The first left-sided source shows a peak in the –500 to 0 ms interval and a trough at roughly 250 ms post-feedback, suggesting that this may be related to movement of the right hand and to the immediate feedback response.

On the right, the first source appears to be related to the feedback response, with a sharp first peak and a damped double-peak structure. The second and third sources show activity primarily in the pre-feedback period, with the second source showing a distinction between the response at 500 ms pre-feedback and at 200 ms pre-feedback, supporting earlier suggestions that within the anticipation period there are different components. The third component shows a prolonged trough during the 500 ms prediction window.

**FIGURE 3:**
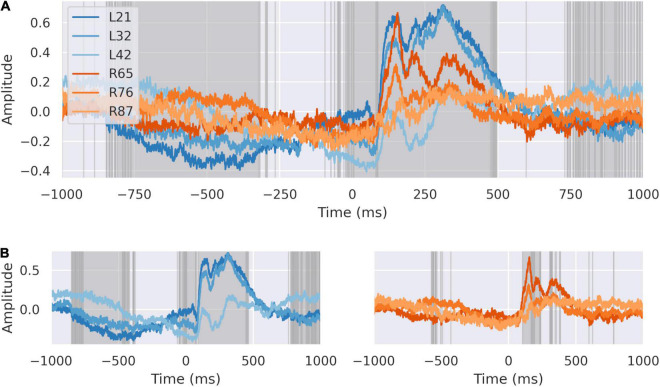
The dACC response is distributed spatially. **(A)** The signals at each electrode plotted together. Highlighted regions demonstrate where differences between electrodes are statistically significant at *p* < 0:0001, Bonferroni corrected. All electrodes show coordinated activity during the –200 ms preparatory window. There are significant differences between individual electrodes in response recorded suggesting different response time courses in discrete populations. **(B)** Average response at left (blue) and right (orange) dACC. Highlighted regions are where the signal on electrodes within that side differ to a statistically significant degree. Individual regions differ in their response to feedback within the intervals we have identified, suggesting a spatial distribution in activity.

We compared the average signal for each bipolar electrode in the left and right dACC (Figure 3). Highlighted regions show the areas where the signals recorded on each electrode on each side differ to a statistically significant degree, based on analysis of variance. We confirmed that the independent components in the averaged signal were spatially distributed within the dACC, evidenced by the morphological and statistical differences in the peri-feedback period in the ERPs from the left and right dACC. Response to feedback and motor-correlated activity were localized to deep areas of the dACC on both sides. All areas synchronized –200 ms before feedback and all areas showed an early response to feedback.

### Network Nodes Communicate at Key Intervals Prior to Feedback and in Response to Feedback Valence

**FIGURE 4:**
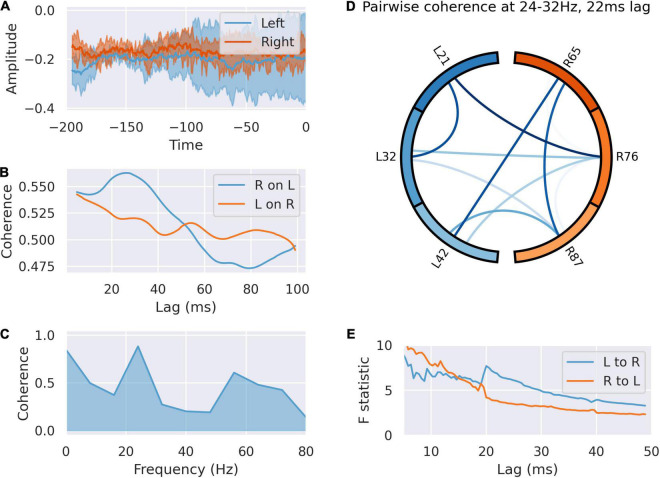
The left dACC signals to the right during the –200 ms preparatory window. **(A)** Averaged responses of the left and right dACC during the –200 ms window. There is a uniform reduction in variance and phase reset across all electrodes here, suggesting coordinated activity. **(B)** Coherence between left and right dACC signals over a range of time lags. Peak coherence occurs between left and right dACC 22 ms later, suggesting left to right communication. **(C)** Coherence spectrum at 22 ms lag. The greatest coherence is seen in the 24–32 Hz range. **(D)** Pairwise coherence for all electrodes at 24–32 Hz with a 22 ms lag. Each area is represented by a labeled part of the external circle (L on the left, R on the right; the numbers indicate the specific electrodes used on the implanted system). The lines joining sections indicate coherence between the joined regions. The darker the color of the connection the greater the coherence between the joined regions. There is widespread coherence during this interval, suggesting coordinated activity. **(E)** Granger causality of signals in this interval. There is a jump in the test statistic at 21 ms, suggesting left to right communication in this interval.

We analyzed communication between right and left in the peri-feedback epoch in light of the laterality of activity between right and left dACC. Coherence analysis and Granger causality indicated bidirectional communication between and within left and right in the –200 ms pre-feedback period in both the alpha/low beta (10–20 Hz) and gamma bands (30–60 Hz), left sided activity preceding right by 20 ms. The dynamics of this communication are not affected by subsequent outcome valence of the trial, agreeing with the decoder accuracy results of Weiss et al. (2018; Figure 4).

**FIGURE 5:**
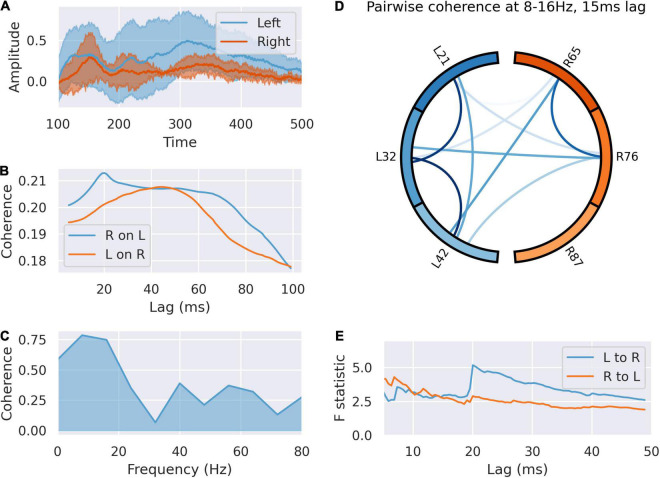
The left signals to the right dACC during the feedback response. **(A)** Averaged responses of left and right dACC during the 100–500 ms feedback response. The right dACC shows a lagged, blunted response, suggesting it is responding to signals from the left ACC rather than inputs directly. **(B)** Coherence between left and right dACC signals over a range of time lags. Peak coherence occurs between left dACC and right 15 ms later, suggesting communication from left to right. **(C)** Coherence spectrum at 15 ms lag. Most of the coherence is accounted for by the sub-16 Hz band. **(D)** Pairwise coherence for all electrodes at 8–16 Hz with a 15 ms lag. There is widespread coherence during this interval, suggesting coordinated activity. **(E)** Granger causality of signals in this interval. There is a jump in the test statistic at 20 ms, suggesting left to right communication in this interval.

**FIGURE 6:**
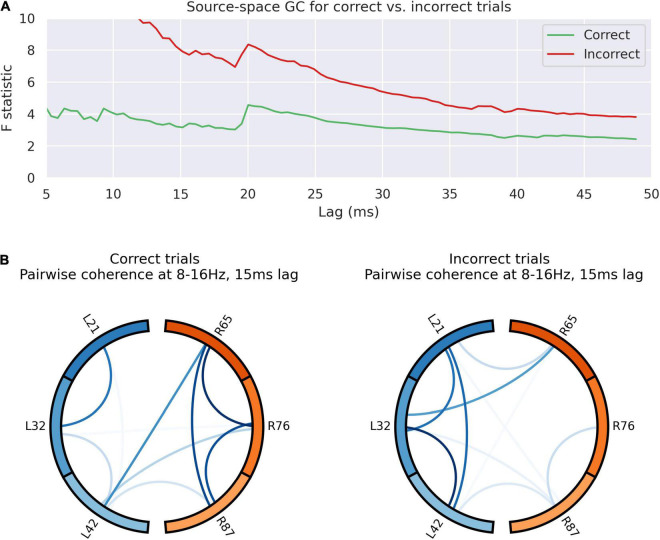
Outcome valence alters information flow in the dACC prediction network. **(A)** Granger causality of activity sources for left to right communication in the 100–500 ms post-feedback window. The test statistic is consistently higher following incorrect trials, suggestive of greater left-to right communication following an error. There is a jump in the test statistic at 20 ms. This suggests transmission of an error-related signal from left to right following incorrect trials with a lag of 20 ms, consistent with other results. The overall high left to right communication seems to be largely driven by incorrect trials. **(B)** Pairwise coherence for all electrodes at 8–16 Hz with a 15 ms lag for correct and incorrect trials. This lag was selected based on the analysis of coherence data across all trials (see Figures 5B,D). The overall patterns of communication are visibly different between trials, as confirmed by principal component analysis (*p* < 0.0001), suggesting different network dynamics based on the nature of the outcome observed.

Post feedback, coherence analysis indicates flow of information from left to right (Figure 5), but the dynamics of this were altered by feedback valence (Figure 6). Granger causality between left dACC and right dACC populations following source decomposition for correct and incorrect trials were compared. For incorrect trials, there was a persistently greater test statistic throughout the lags, indicating greater left-to-right information flow following negative feedback. This indicates that the overall high left to right communication seen in Figure 5E is largely driven by the response to incorrect trials, where a high left-to-right information flow is present. This then gradually drops off, although there is a clear step in the test statistic; this occurs at 21 ms (*F* = 5.83; *p* < 0.0001 ÷ 100). This is consistent with the lag at which we have previously shown communication within the dACC network above (Figure 4). Notably, this is still present, though at a lower amplitude, during correct trials.

Figure 6 indicates graphically the pair-wise connectivity between electrodes in following correct and incorrect feedback. This indicates the contribution of spatial nodes to the network response to outcome valence. This was statistically compared by decomposing the matrix of pairwise coherence measures into its principal components, identifying the component that accounts for the majority of the variance in network architecture and comparing this. There is a statistically significant overall difference in network connectivity based on outcome valence (*p* < 0.0001).

**FIGURE 7:**
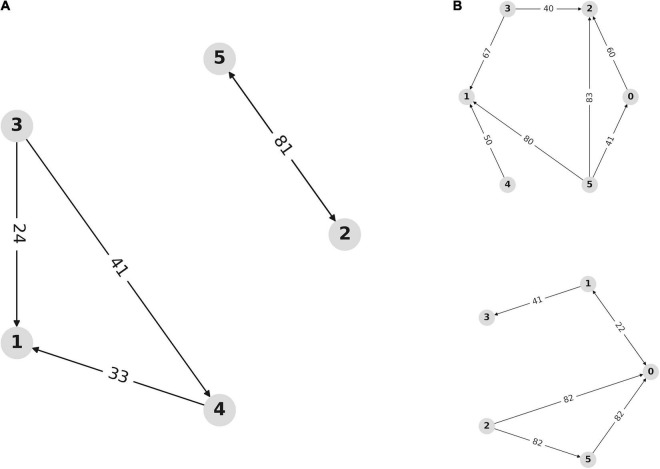
Network dynamics are affected by outcome valence. **(A)** Network inferred from multivariate transfer entropy of independent activity sources for all trials. Nodes represent sources within the dACC network. Arrows represent the direction of information transfer between populations. Numbers indicate the lag (in samples at 2048 Hz) at which communication occurs between network nodes. This allows us to visualize dynamics within the dACC prediction network. Nodes 0, 1, and 2 are located in the left ACC with nodes 3, 4, and 5 on the right. Nodes 1, 3, and 4 consistently interact, as do nodes 2 and 5, but these subnetworks do not consistently communicate. **(B)** Network inference using multivariate transfer entropy of independent sources during correct and incorrect trials. Outcome valence results in an alteration in network dynamics through alterations in the temporal characteristics of information flow within the dACC prediction network. The top graph shows the network inferred from correct trials, while the bottom graph shows the network inferred from incorrect trials. The pattern of network communication differs based on outcome valence.

Multivariate transfer entropy offers another method of analyzing network dynamics that is inherently multivariate and avoids assumptions about the nature of the underlying network dynamics. Figure 7 demonstrates the network inferred from analyses of transfer entropy between the source-space representations of the overall signal. This represents the temporal dynamics of information flow between the neuronal populations within the dACC network.

Figure 7A shows that activity sources form directional networks with time lags that are physiologically plausible and within the range described above, supporting the hypothesis that the dACC contains an interacting network of populations that predict and respond to outcomes. Figure 7B shows the inferred network for correct (above) and incorrect (below) trials. Network dynamics within the dACC differ based on outcome valence, indicating that information flow within the dACC predictive network is modulated by outcome valence.

### Left and Right Dorsal Anterior Cingulate Cortex Response to New Stimulus Is Not Influenced by Outcome Valence of the Previous Trial

**FIGURE 8:**
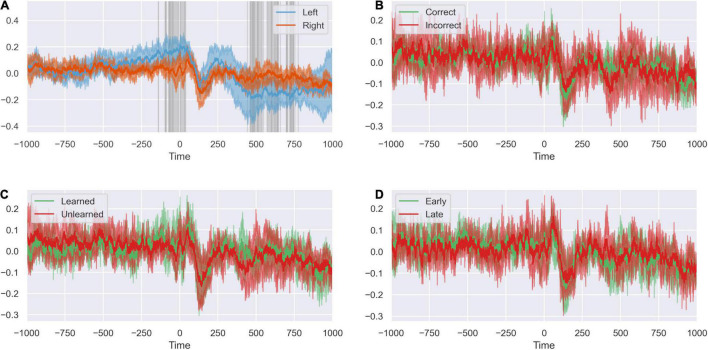
The peri-stimulus response is not modified by task variables. **(A)** Response of left and right ACC time-locked to stimulus presentation. Highlighted regions show where activity differs between sides. There is event of peri-stimulus activity in the ACC. Differences in activity between sides indicates lateralization of the stimulus response. **(B)** The peri-stimulus response of the ACC when the previous trial was correct and incorrect. The outcome of the previous trial does not appear to influence the stimulus response on subsequent trials. **(C)** The peri-stimulus response of the ACC when the task has been learned or unlearned. Whether the present task has been learned does not appear to influence the stimulus response. **(D)** The peri-stimulus response of the ACC for early and late trials. The peri-stimulus response does not appear to differ between early and late trials.

Having established the dynamics of the response to feedback of the preceding trial, we investigated how the network responds at new stimulus presentation in the subsequent trial (Figure 8). We again saw a difference between left and right dACC. Left dACC anticipated a new stimulus between blank boxes appearing on screen at the start of the trial and presentation of the actual stimulus (–200 ms to stimulus presentation at 0 ms). Both hemispheres responded with a simultaneous event related potential (0 to +250 ms) followed by the feedback anticipation signal described above in the left dACC (Figure 8). However, we did not detect a significant difference between right or left dACC response between positive and negative feedback (Supplementary Figure 4).

We compared response of right and left dACC to factors we suspected might influence the dACC response to stimuli presentation after a previous error: familiarity with the stimulus and whether empirically the subject had learned the underlying rule governing object choice in the task. We found no significant differences between right and left dACC in unlearned rule trials compared to learned rule trials nor trials early in a rule compare to late in a rule (Supplementary Figure 4).

### Learning Modifies Communication Between Left and Right Dorsal Anterior Cingulate Cortex

To compare the behavior of the dACC network in trials when our subjects had learned versus had not learned the underlying rule, we analyzed both directional and functional connectivity in low (30–60 Hz) and high (60–100 Hz) gamma, beta (12.5–30 Hz) and theta (4–8 Hz) frequency ranges.

We calculated the difference in directional connectivity between the recording contacts within the left and right dACC as well as across hemispheres. The latter was done to assess evidence of interhemispheric cross-talk and investigate further any prior observations pointing toward asymmetry/laterality in information processing.

With regards to coherence, the analysis of the entire connectivity matrix revealed a significant difference only in the low gamma (30–60 Hz) range – with a decrease in coherence between the left and right dACC in the learned trials (*p* = 0.0413 CI: [–0.0364 –0.0007]) (Table 1). When the average left-to-right dACC cross-talk was evaluated separately, an increase in coherence between the left and right dACC in the beta range was also revealed (*p* = 1.7697 × 10-13, CI [0.0062 0.0107]) (Table 1).

**TABLE 1:** Inter- and intra-hemispheric functional connectivity, as measured by magnitude-squared coherence.

Frequency range	Mean learned	Mean unlearned	*p*-value	Conf. interval
**Coherence –cross-talk between hemispheres**				
Low gamma	0.0553	0.0723	0.0000	[–0.0238 –0.0101]
High gamma	0.0448	0.0514	0.0854	[–0.0139 0.0009]
Beta	0.0444	0.0360	0.0000	[0.0062 0.0107]
Theta	0.0921	0.0975	0.2771	[–0.0152 0.0044]
**Coherence – all contact combinations**				
Low gamma	0.2404	0.2589	0.0413	[–0.0364 –0.0007]
High gamma	0.2802	0.2677	0.1274	[–0.0036 0.0285]
Beta	0.2571	0.2477	0.4341	[–0.0142 0.0331]
Theta	0.3219	0.3107	0.6459	[–0.0365 0.0588]

Learning was also associated with statistically significant increases in directional connectivity in the theta, beta and gamma range. This was true for both the connectivity matrix of all recording contact combinations (local activity and cross-talk), as well as for cross-talk between left and right dACC (Supplementary Table 1). These levels of significance persisted for changes in model order.

## Discussion

We demonstrate a temporally and spatially distributed cognitive network between dominant (left) and non-dominant (right) hemispheres in the dACC that anticipates and responds contingently to feedback within an area associated with internally generated movement and pain function (Misra and Coombes, 2015; Oane et al., 2020). The functional network information flow is mainly asymmetric since information flows predominantly from left (dominant) to right (non-dominant) after feedback signaling the outcome of the voluntary movement in these right-handed subjects. This information flow is altered by outcome valence and is modified by learning.

The functional heterogeneity of the ACC is well described (Behrens et al., 2007), but this data provides evidence that cognitive functions including anticipation, prediction error and learning are embedded spatially with pain and motor functions in humans. Our results further elucidate the functional-anatomical arrangement of the human dACC and the asymmetric dynamics of right-left information flow during real-time behavior within the human dACC network.

### Early Anticipation

We show here a negative going potential in the left dACC preceding feedback by 500 ms. This was temporally associated with right arm movement to the touch screen hosting the task but not present in the right dACC. On the basis of fMRI and stimulation studies the dACC has been ascribed a motor function in humans (Hoffstaedter et al., 2014; Oane et al., 2020), specifically for internally generated movements, including contralateral upper limb reaching and grasping movements that are relevant in this task (Caruana et al., 2018). The movement-associated activity in the left dACC shown here may potentially represent its participation in internally generated movement, which is supported by its strong diffusion tensor MR imaging connectivity with the supplementary motor area (Weiss et al., 2018). Indeed, subpopulations of dACC neurons are active during the motor execution phase of the task, particularly when behavioral flexibility is required (Procyk et al., 2000). In addition to action selection, the dACC participates in the initiation of movements more generally (Srinivasan et al., 2013), which may explain why this pre-feedback phase was invariant to subsequent feedback valence. Alternatively, there may be a non-motor contribution to this anticipatory potential, such as has been reported in the human EEG literature (Kotani et al., 2015). While difficult to disentangle with the present experimental paradigm, the laterality of our finding may be more suggestive of motor activity. However, future work systematically varying the motor element of the task is warranted to further explore this task phase.

### Late Anticipation

We demonstrate a reliable anticipatory response evident in both the right and left dACC 200ms before the onset of feedback. All electrodes synchronize their activity bilaterally. The dynamics of communication in this phase are the same whether the feedback subsequently signals correct or incorrect choice. A similar pre-stimulus activity was seen in the left dACC before new stimulus presentation. In the case of predictable, rhythmic visual (Lakatos et al., 2008) and auditory (Stefanics et al., 2010) cues it has been suggested that anticipatory phase-entrainment responses improve performance. Such phase entrainment provides greater sensitivity to incoming stimuli, and allows the system to react rapidly for the optimal processing of information (Schroeder and Lakatos, 2009). Anticipatory phase-entrainment responses are also potentiated by selective attention toward attributes of the predicted stimulus, such as its spatial location (Zareian et al., 2020). Our finding of a coordinated phase reset before feedback likely suggests the dACC is making or participating in a network that predicts the occurrence of sensory feedback in response to movement, but not the valence of the feedback.

### Response to Feedback

The dACC network exhibits a spatially and temporally distributed response to feedback, contingent on the valence of the feedback. Incorrect feedback results in a statistically significantly greater response at 200–500 ms in the left dACC, confirming (Weiss et al., 2018). The timing and overall response profile of these components were generally consistent with the prediction error responses that have been reported in human EEG studies using a similar reward-learning task (Philiastides et al., 2010; Fouragnan et al., 2015, 2017). The timing of the early and late components corresponded to a single theta oscillation period, supporting a large body of evidence implicating frontal theta in feedback-mediated behavioral adjustment (Klimesch, 1999; Womelsdorf et al., 2010a; Cavanagh and Frank, 2014).

Our results complement prior evidence from EEG-informed fMRI (Hauser et al., 2014) and invasive recordings in non-human primates localizing frontal midline theta to the dACC. Results from the latter have shown associations between increased theta power and phasic coupling of single neuronal spikes within the theta range, suggesting the creation of a temporal window role to allow information transfer leading to behavioral adjustment (Womelsdorf et al., 2010a). This window has been suggested to reflect the co-ordination of inputs across cortical areas, allowing integration of choice-relevant information such as stimulus-response mapping rules, context, and reward in order to facilitate action selection (Womelsdorf et al., 2010b).

Our findings are complementary to these results, showing the generation of a theta oscillation when the task rule needs to be updated and an adjustment to action selection is required. This task-related information transfer through theta-band phase synchrony has been observed between the dACC and a variety of cortical sites (reviewed in Cavanagh and Frank, 2014). While the above studies have communicated trial outcomes with auditory or visual feedback, it is notable that human microelectrode recordings have suggested similar dACC responses to self-detected error (Wang et al., 2005). Additionally, studies systematically manipulating feedback have shown increased (negative valence) feedback reliability is associated with greater frontal midline theta power and behavioral adjustment (Li et al., 2018). While the task in the current study presented auditory feedback at regular intervals, it is important for future work to explore the variations in dACC response to differing sensory modalities, reliability and magnitude of feedback.

### Learning Affects Interhemispheric Gamma Synchrony Between Left and Right Dorsal Anterior Cingulate Cortex

Our categories learned/unlearned and early/late trials were attempts to examine the effect of different cognitive dimensions on dACC function. We were not able to investigate the electrophysiological correlates of other cognitive dimensions of reward-based decision making as we were unable to collect information on this during this real time task. This fact is of particular importance when taking into consideration our finding with regards to decrease in low-gamma coherence, since reward probability is also reportedly encoded in dACC activity at this frequency, based on recent work in rodents (Boroujeni et al., 2021). Based on other prior published evidence, gamma synchrony has been shown to be required when reappraisal of behavioral salience of familiar external cues is required – and is the result of the activity of discrete interneuronal populations in the dACC and prefrontal cortex.

Functional connectivity analyses and recording parameters used here cannot be presumed to be a direct reflection of discrete anatomical networks or compared to the level of accuracy of micro-electrode or single-cell recordings. In addition, a high level of cytoarchitectural differentiation has been reported in the dACC using such methods (Vogt and Paxinos, 2014; Boroujeni et al., 2021)– it would be reasonable to assume that our recording samples different equivalent sub-regions with a variety of cellular populations. However, our results that gamma coherence (30–60 Hz) decreases in learned trials in comparison to unlearned (where cue reappraisal is required) seems to be a direct translation of recent rodent model findings where parvalbumin interneuron activity was predominantly noted in similar scenarios. With regards to directional underpinnings of this relationship, our results are less clear – involving a wider range of frequencies, which could reflect the population heterogeneity sampled during our experiments (Cho et al., 2020).

### Dorsal Anterior Cingulate Cortex Network Responds to Task Performance

We show that dACC network dynamics respond to outcome valence. We further show that network dynamics are affected by rule learning. We observed that learning a rule was associated with greater connectivity between right and left dACC at physiologically relevant frequencies (Cohen, 2016; Adams et al., 2017). This suggests outcome valence information of actions over repeated trials directly or indirectly influences information flow in the dACC network. This may represent an electrophysiological manifestation of working memory, although whether memory is represented locally or projected from other regions is not clear from our data (Miller et al., 2018). This may shed light on how reinforcement learning occurs at cellular level in human subjects: the representation of predictions concerning actions likely to yield positive outcomes in relation to the rule, in this case correct object selection (Akam et al., 2021).

However, despite a robust response to error demonstrated at feedback, we did not detect in our recordings an influence of error on response to new stimulus. This is in contrast to findings in the macaque that indicate a role for the ACC in stimulus response mapping at object presentation after error (Womelsdorf et al., 2010a). This may be due to the relative size of the macaque and human anterior cingulate cortex compared to the volume of tissue recorded by DBS electrodes (Lempka and McIntyre, 2013) resulting in recordings from functionally distinct regions. Parcelation of the ACC in humans and macaques is well described suggesting distinct spatially localized functional areas of the ACC (Sallet et al., 2013; Jin et al., 2018; Margulies and Uddin, 2019; Palomero-Gallagher et al., 2019).

Moreover our independent component analysis appeared to reveal components of the dACC response could be spatially localized to individual bipolar recordings. Our recordings represent the activity of a small localized area of a few mm^3^ within the dorsal ACC which may not reflect the proportionately larger area recorded in Womelsdorf, which could represent the activity of more than one parcelation or group of functional nodes of the ACC (Womelsdorf et al., 2010b; Lempka and McIntyre, 2013).

Alternative explanations include that the event-related activity of the right dACC has a variable latency, unlike the feedback ERPs of right and left or the new stimulus ERP of the left dACC (Ouyang et al., 2015). Signal averaging such an ERP may potentially mask information, although we did not see an effect of previous error on timing of the right dACC stimulus ERP when analyzed individually (Supplementary Figure 4).

These conflicting results may also be due to differences in the nature of the tasks used. Factors that could be related to dACC function in decision-making include the influence of surprise outcomes (Fouragnan et al., 2017), salience of feedback or stimuli (Raver and Lin, 2015) or degree of uncertainty/confidence in decision-making (Stolyarova et al., 2019). This would be a key element of future work in these subjects to elucidate the nature of the right dACC stimulus presentation event related potential reported in Weiss et al. (2018).

### Limitations

The present work relies on the application of statistical measures of the relationship between activity levels in brain regions to identify patterns of functional connectivity and how these dynamics are altered by a cognitive task. However, as with any work that examines network dynamics using functional connectivity measures, it must be borne in mind that a statistical relationship between activity levels in two regions does not imply the existence of any anatomical connection between the two. The present study aims to use a set of undirected and directed measures of the relationships between activity measures which are valid under different assumptions; these measures converge on a model where nodes within the ACC communicate with each other with a specific spatial and temporal pattern and that these dynamics are altered by factors such as response salience, implying that these factors affect task-related processing. We cannot, however, make any claims about the underlying structural connections producing this functional relationship.

Furthermore, the possibility that some of these effects are influenced by residual volume conduction from closely adjacent anatomical structures still exists. Further investigations using the imaginary component of coherence could provide more conservative estimates to that extent.

Recordings were normalized within trials in order to facilitate comparisons between individuals and within individuals across recording sessions (Supplementary Figure 5). This reduced the effect of variability in the impedance of the electrode-tissue interface on the amplitude of the recorded local field potential signal and allows consistency across recording sessions. However, where there is event-related activity, this can result in reduction of small event-related changes as larger activity increases the variance of the signal. While this approach allows identification of significant event-related activity as shown here, more subtle event-related changes may not be detected. There may, therefore, be additional feedback-related dynamics within the ACC to those identified in the present work.

Our analysis of the neural response to task variables used independent component analysis to identify independent sources in the data. This allowed us to characterize the response to task events by decomposing it into a set of independent responses. This is a commonly used approach to identify neural populations, or individual neurons in the context of spike sorting, based on the assumption that individual populations mediate a single, characteristic response. However, it is important to note that this is not necessarily true. It is possible for a single neural population to mediate two statistically independent responses. Some apparent networks effects may, therefore, involve multiple responses mediated by the same multi-functional anatomical population. This further underlines the importance of interpreting functional connectivity relationships as purely functional, and not necessarily reflective of the underlying anatomical connections.

Excluded trials were replaced with the mean of all other trials in order to maintain a balanced dataset for within-subjects comparisons between conditions. This is a relatively conservative method for interpolation of excluded trials as it tends to reduce inter-group differences by replacing trials in each condition with the mean over all conditions. In this sense, it is unlikely to bias the results of between-groups comparisons. However, this approach also reduces the variance in the overall dataset. In order to ensure that this interpolation strategy did not influence the results, all analyses were repeated without interpolation of excluded trials. Some of these results are shown in Supplementary Figure 6. There were no differences in any of the measures investigated between the data with and without interpolated trials, indicating that this did not introduce bias, while also allowing balanced between-group comparisons to be performed.

The results presented are all at the population level, representing average network dynamics across subjects. Weiss et al. (2018) have previously demonstrated the consistency of ACC responses across individuals. Further, the features of ACC network dynamics and their responses to task variables demonstrated are also valid at the level of individual subjects. All individual subjects demonstrate consistent ACC dynamics. Supplementary Figure 5 shows the pattern of information transfer from left to right following feedback at the individual subject level, with each participant demonstrating the same dynamics that were evident at the group level. Further analysis shows that this response is driven by a valence-dependent effect, where there is a large left-to-right information flow following incorrect feedback, as is evident at the population level. Our group-level analysis is therefore firmly supported by the individual-level network dynamics. However, the small sample size present here means that despite these results, a larger sample size and further tasks will be required to further elucidate the task-dependent dynamics of the ACC in general.

## Conclusion

These results suggest that cognition is embedded in the dACC, temporally and spatially distributed between nodes located within and between the two hemispheres. Information flow between right and left dACC is predominantly asymmetric: the left dACC responds to movement; both right and left anticipate the receipt of sensory feedback triggered by the movement; and left dACC signals error to right dACC with a time delay of 15–20 ms. Learning of a rule alters the connectivity of the network, potentially in a manner relevant to uncertainty resolution reflected in gamma-synchrony. Unexpectedly, however, we did not observe an effect of previous error on new stimulus presentation response. We show that these cognitive functions are spatially distributed within the area of the dACC that is targeted by deep brain stimulation to treat the affective component of neuropathic pain and that participates in generating internally driven voluntary movement with the supplementary motor area. Our findings suggest that cognitive function is both temporally and spatially distributed and embedded with both pain perception and motor function in the dACC.

## Data Availability Statement

The data analyzed in this study is subject to the following licenses/restrictions: the data presented here are available from the corresponding author on reasonable request. Analysis code is available at https://github.com/Oxford-Functional-Neurosurgery/ACC-processing. Requests to access these datasets should be directed to MG, martin.gillies@nds.ox.ac.uk.

## Ethics Statement

This study was carried out in accordance with the recommendations and approval of Oxfordshire Research Ethics Committee A (Ref 11/SC/0229). All subjects gave written informed consent in accordance with the Declaration of Helsinki. The patients/participants provided their written informed consent to participate in this study.

## Author Contributions

AW and MG collected the data previously and prepared the data for analysis in this manuscript. CK, MZ, AD, and CS designed and performed the data analysis. MZ, AZ, MP, SB-B, and JS wrote and edited the manuscript. JF, AG, and TA provided supervision and funding of the project. MG directed the project. All the authors contributed to the article and approved the submitted version.

## Conflict of Interest

The authors declare that the research was conducted in the absence of any commercial or financial relationships that could be construed as a potential conflict of interest.

## Publisher’s Note

All claims expressed in this article are solely those of the authors and do not necessarily represent those of their affiliated organizations, or those of the publisher, the editors and the reviewers. Any product that may be evaluated in this article, or claim that may be made by its manufacturer, is not guaranteed or endorsed by the publisher.

## Supplementary Material

Supplementary Figure 1 Deep brain stimulation electrode contact locations in the subject space. Postoperative computed tomography (CT) scans were registered to preoperative structural T2-weighted MRI images using FLIRT^1^, allowing electrode positions to be plotted in each subject’s structural space. Panels A-C show sequential 1 mm T2 axial slices representing patients 1–3, respectively, with superimposed electrode contact positions. C0 (red) represents the ventral-most contact, and C3 (yellow) the dorsal-most contact, with C1 (cyan) and C2 (green) the middle two contacts. Electrodes were satisfactorily placed in the dorsal anterior cingulate cortices, corroborating MNI-space registrations in Weiss et al. (2018).

Supplementary Figure 2 Intra- extradimensional set shift task. **(A)** Schematic of the task from the Cambridge Neurophysiological Test Automated Battery (CANTAB) displaying rule order during a given recording. Green rectangles indicate the correct choice. The rule progresses after six consecutive trials with correct responses. The test terminates if six consecutive correct trials cannot be made over a period of 50 attempts. Copyright 2018 Cambridge Cognition, Ltd. All rights reserved. **(B)** Schematic representation of sensory and motor events within a given trial. A trial begins with the presentation of two visual, abstract objects. After a variable-length decision-making phase, the subject then makes a movement to touch the CANTAB test screen with their dominant hand. A screen press elicits auditory and visual feedback indicating whether the subject has chosen the correct or incorrect figure for the current rule. After an interval of 1.5 s, the screen turns blank and then begins the next trial. Reproduced with permission from Figure 1 of Gillies et al. (2017), under the open access Creative Commons Attribution 4.0 International License.

Supplementary Figure 3 Principal component weights. Visualizations of the weights of the first three principal components of network communication over the pairwise connectivity metrics. The first component appears to indicate global network activity; the second and third components appear to show some selectivity for particular patterns of activity. These components are used to provide a global assessment of patterns of network communication and how these differ with task variables.

Supplementary Figure 4 The peri-stimulus response within each side is not modified by task variables. **(A)** There is no difference in the stimulus response within the right ACC when the previous trial was correct or incorrect. **(B)** There is no difference in the stimulus response within the right ACC when the current task is learned or unlearned. **(C)** There is no difference in the stimulus response within the right ACC when the current trial is early or late. **(D)** There is no difference in the stimulus response within the left ACC when the previous trial was correct or incorrect. **(E)** There is no difference in the stimulus response within the left ACC when the current task is learned or unlearned. **(F)** There is no difference in the stimulus response within the left ACC when the current trial is early or late.

Supplementary Figure 5 Individual-level information transfer. Granger causality measures for left-to-right and right-to-left information flow following feedback for each individual participant. The directionality of information flow following feedback observed on the group is level is consistently present within each individual. There is a greater level of left-to-right information flow with a jump in the test statistic at roughly 20 ms following feedback. Results observed at the group level are consistent across subjects at the individual level.

Supplementary Figure 6 Interpolation of excluded trials does not bias results. **(A)** The average feedback-locked response across all channels; highlighted regions demonstrates where the response is statistically significant. There are no changes compared to the analysis in Figure 1A. **(B)** Power spectral density and phase spectrum across all channels, time-locked to feedback. There are no changes compared to the analysis in Figure 1B. **(C)** Response of left and right dACC. Highlighted regions show where activity is significantly different between sides. There are no changes compared to the analysis in Figure 2A. **(D)** Averaged responses, coherence and coherence spectrum between right and left ACC during the –200 ms preparatory window. There are no changes compared to the analyses in Figures 4A–C. **(E)** Pairwise coherence for all electrodes at 24–32 Hz with a 22 ms lag. Each area is represented by a labeled part of the external circle (L on the left, R on the right; the numbers indicate the specific electrodes used on the implanted system). The lines joining sections indicate coherence between the joined regions. The darker the color of the connection the greater the coherence between the joined regions. There are no changes compared to the analysis in Figure 4D. **(F)** Averaged responses, coherence and coherence spectrum between right and left ACC during the 100–500 ms feedback window. There are no changes compared to the analyses in Figures 5A–C. **(G)** Pairwise coherence for all electrodes at 8–16 Hz with a 15 ms lag. There are no changes compared to the analysis in Figure 5D.
