# Supplementary material for: Spatial and Temporal Distribution of Information Processing in the Human Dorsal Anterior Cingulate Cortex
Source: Front Hum Neurosci. 2022 Mar 18;16:780047. doi: 10.3389/fnhum.2022.780047 (PMC8973009; doi:10.3389/fnhum.2022.780047)
Supplement: Supplementary file 1 [file Table_1.DOCX]

| **Directional Connectivity –all contact combinations** | | | | |
| --- | --- | --- | --- | --- |
| **Frequency range** | **p-value** | **Conf. Interval** | **Mean Learnt** | **Mean Unlearnt** |
| **Low Gamma** | 0.0000 | [0.0087 0.0173] | 0.0515 | 0.0385 |
| **High Gamma** | 0.0000 | [0.0071 0.0124] | 0.0438 | 0.0340 |
| **Beta** | 0.0000 | [0.0113 0.0280] | 0.0698 | 0.0502 |
| **Theta** | 0.0162 | [0.0033 0.0324] | 0.0629 | 0.0450 |

| **Directional Connectivity –cross-talk between hemispheres** | | | | |
| --- | --- | --- | --- | --- |
| **Frequency range** | **p-value** | **Conf. Interval** | **Mean Learnt** | **Mean Unlearnt** |
| **Low Gamma** | 0.0275 | [0.0000 0.0008] | 0.0045 | 0.0040 |
| **High Gamma** | 0.0000 | [0.0032 0.0218] | 0.0218 | 0.0173 |
| **Beta** | 0.0000 | [0.0016 0.0024] | 0.0046 | 0.0026 |
| **Theta** | 0.0001 | [0.0001 0.0018] | 0.0122 | 0.0085 |

Supplementary Table 1. Directional connectivity for inter- and intra-hemispheric interactions, as estimated by GC.
