# Supplementary figures and images for: Spatial and Temporal Distribution of Information Processing in the Human Dorsal Anterior Cingulate Cortex
Source: Front Hum Neurosci. 2022 Mar 18;16:780047. doi: 10.3389/fnhum.2022.780047 (PMC8973009; doi:10.3389/fnhum.2022.780047)

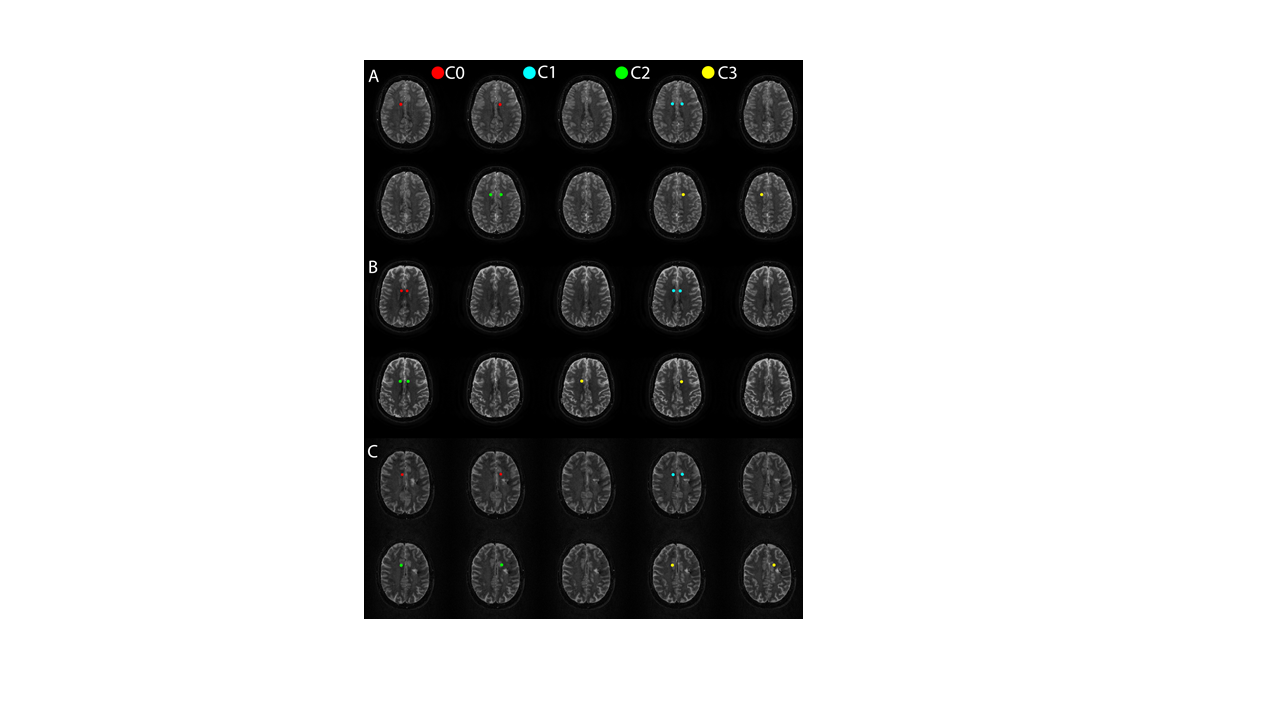

Supplement: Supplementary Figure 1 — Deep brain stimulation electrode contact locations in the subject space. Postoperative computed tomography (CT) scans were registered to preoperative structural T2-weighted MRI images using FLIRT1, allowing electrode positions to be plotted in each subject’s structural space. Panels A-C show sequential 1 mm T2 axial slices representing patients 1–3, respectively, with superimposed electrode contact positions. C0 (red) represents the ventral-most contact, and C3 (yellow) the dorsal-most contact, with C1 (cyan) and C2 (green) the middle two contacts. Electrodes were satisfactorily placed in the dorsal anterior cingulate cortices, corroborating MNI-space registrations in Weiss et al. (2018). [file Image_1.TIF]

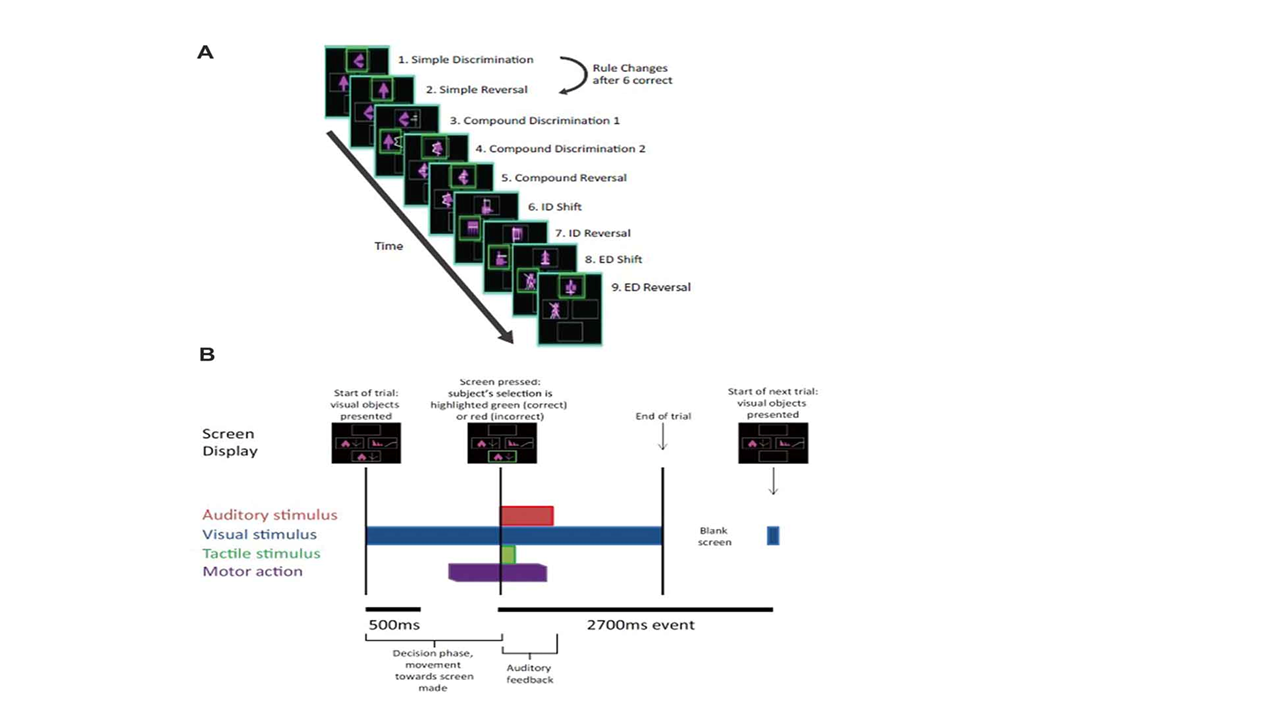

Supplement: Supplementary Figure 2 — Intra- extradimensional set shift task. (A) Schematic of the task from the Cambridge Neurophysiological Test Automated Battery (CANTAB) displaying rule order during a given recording. Green rectangles indicate the correct choice. The rule progresses after six consecutive trials with correct responses. The test terminates if six consecutive correct trials cannot be made over a period of 50 attempts. Copyright 2018 Cambridge Cognition, Ltd. All rights reserved. (B) Schematic representation of sensory and motor events within a given trial. A trial begins with the presentation of two visual, abstract objects. After a variable-length decision-making phase, the subject then makes a movement to touch the CANTAB test screen with their dominant hand. A screen press elicits auditory and visual feedback indicating whether the subject has chosen the correct or incorrect figure for the current rule. After an interval of 1.5 s, the screen turns blank and then begins the next trial. Reproduced with permission from Figure 1 of Gillies et al. (2017), under the open access Creative Commons Attribution 4.0 International License. [file Image_2.TIF]

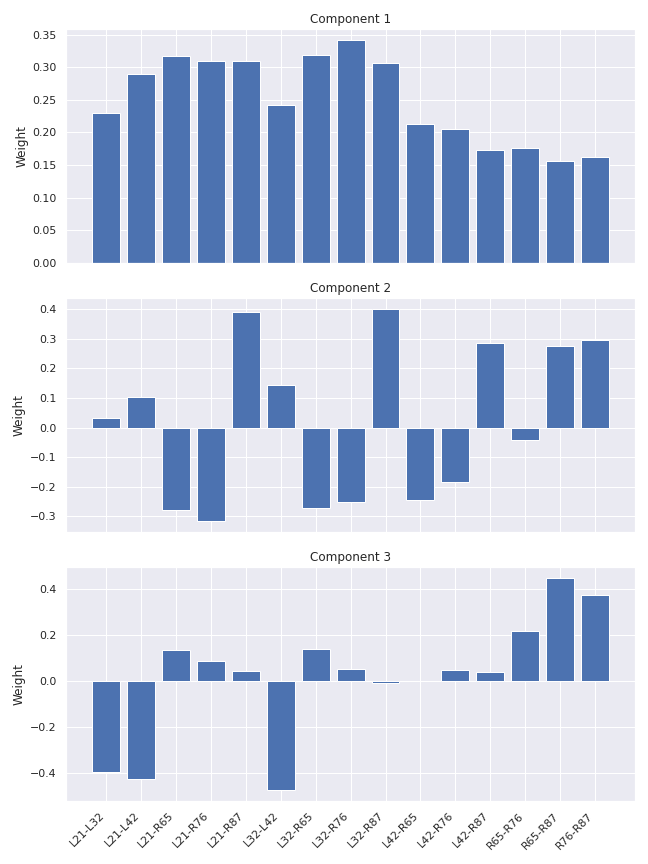

Supplement: Supplementary Figure 3 — Principal component weights. Visualizations of the weights of the first three principal components of network communication over the pairwise connectivity metrics. The first component appears to indicate global network activity; the second and third components appear to show some selectivity for particular patterns of activity. These components are used to provide a global assessment of patterns of network communication and how these differ with task variables. [file Image_3.TIFF]

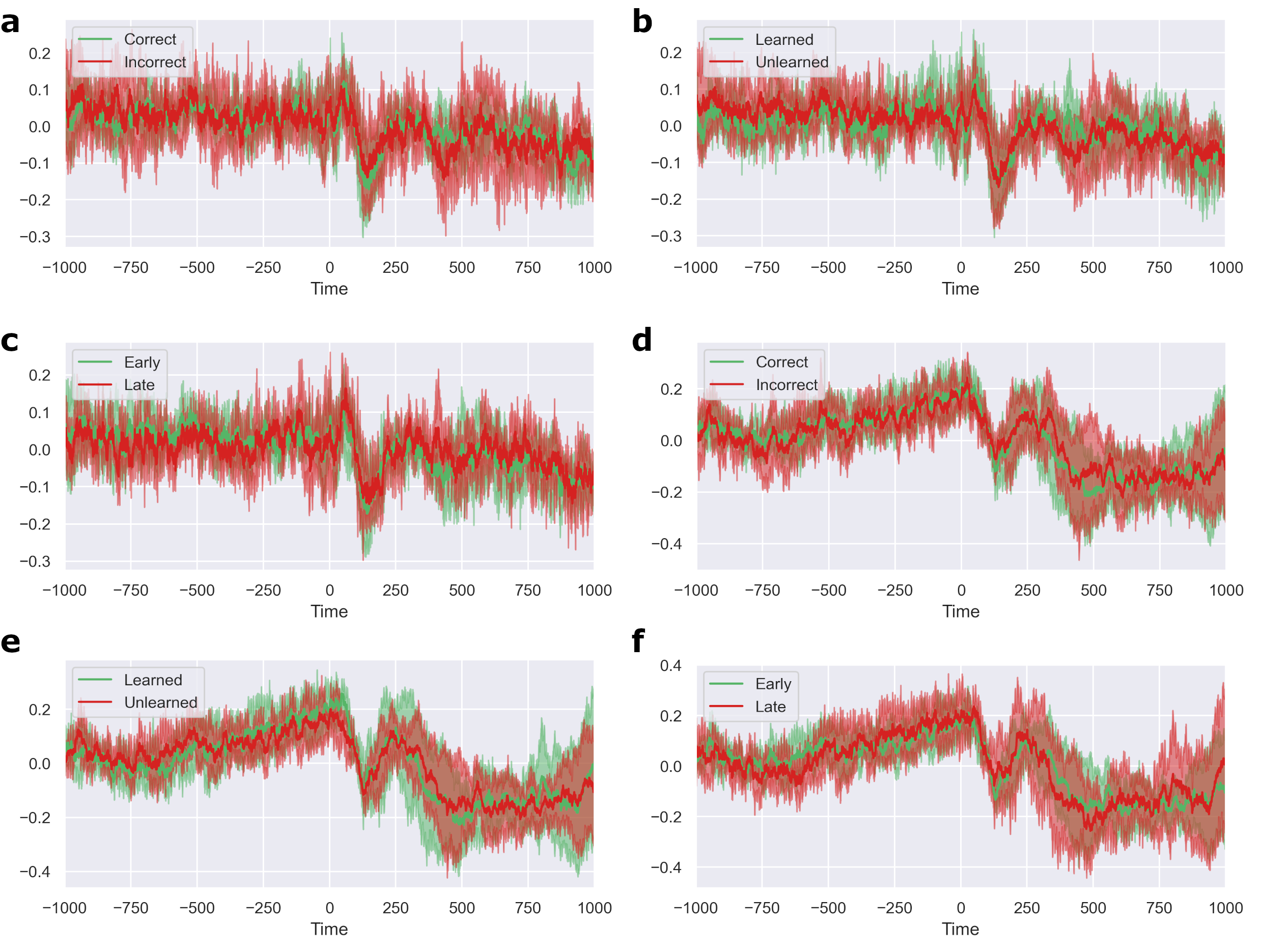

Supplement: Supplementary Figure 4 — The peri-stimulus response within each side is not modified by task variables. (A) There is no difference in the stimulus response within the right ACC when the previous trial was correct or incorrect. (B) There is no difference in the stimulus response within the right ACC when the current task is learned or unlearned. (C) There is no difference in the stimulus response within the right ACC when the current trial is early or late. (D) There is no difference in the stimulus response within the left ACC when the previous trial was correct or incorrect. (E) There is no difference in the stimulus response within the left ACC when the current task is learned or unlearned. (F) There is no difference in the stimulus response within the left ACC when the current trial is early or late. [file Image_4.TIFF]

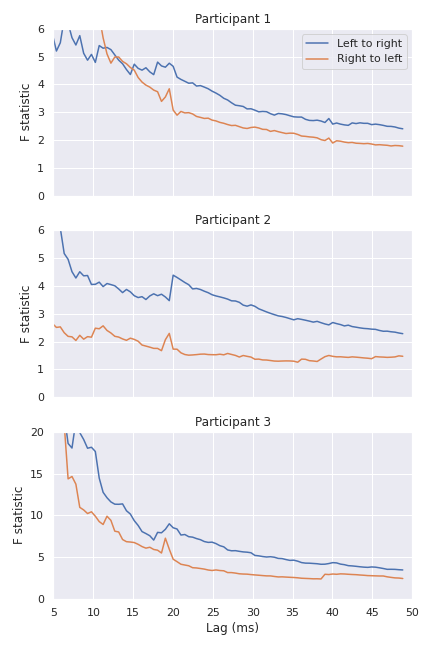

Supplement: Supplementary Figure 5 — Individual-level information transfer. Granger causality measures for left-to-right and right-to-left information flow following feedback for each individual participant. The directionality of information flow following feedback observed on the group is level is consistently present within each individual. There is a greater level of left-to-right information flow with a jump in the test statistic at roughly 20 ms following feedback. Results observed at the group level are consistent across subjects at the individual level. [file Image_5.TIFF]

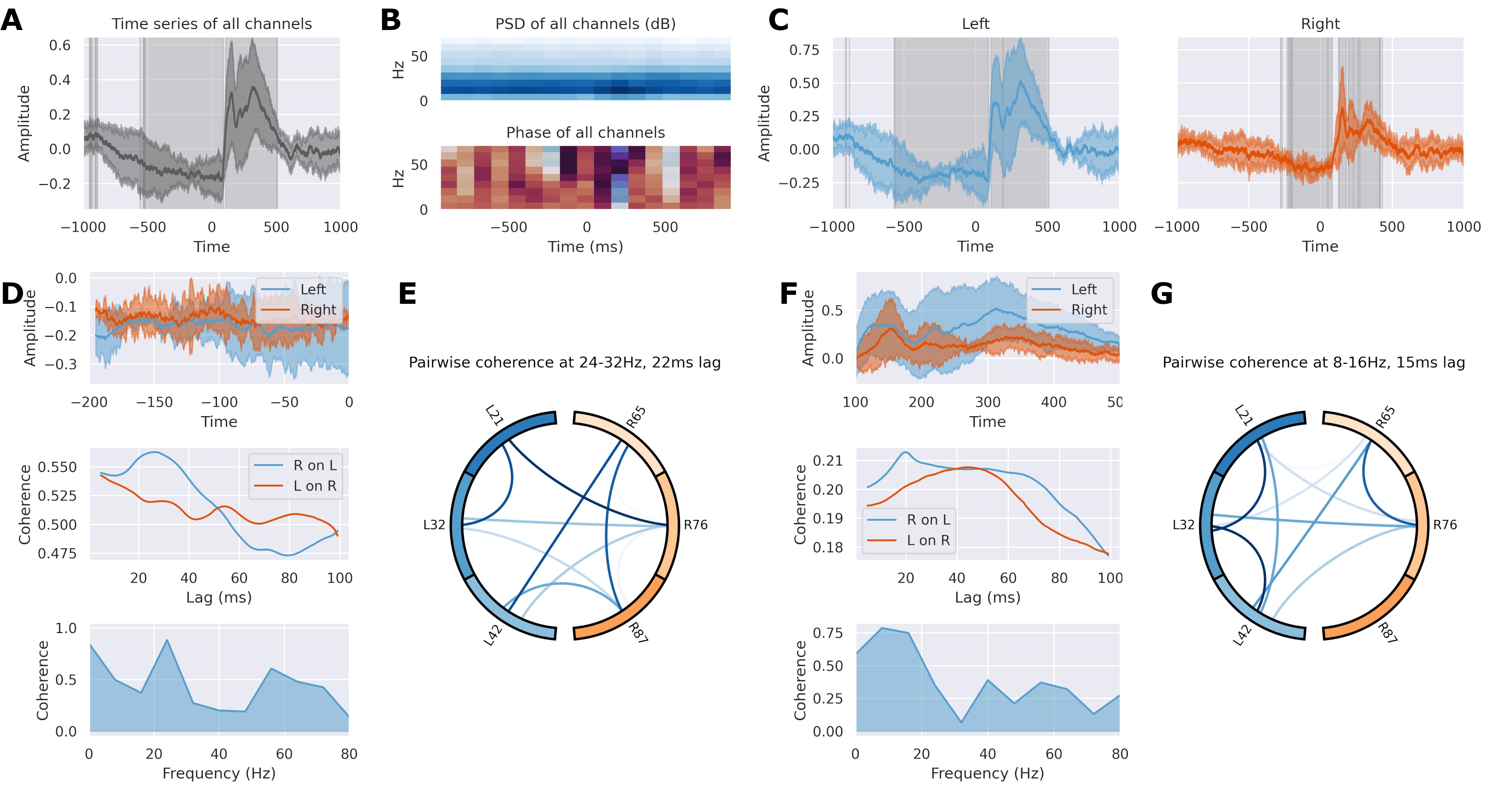

Supplement: Supplementary Figure 6 — Interpolation of excluded trials does not bias results. (A) The average feedback-locked response across all channels; highlighted regions demonstrates where the response is statistically significant. There are no changes compared to the analysis in Figure 1A. (B) Power spectral density and phase spectrum across all channels, time-locked to feedback. There are no changes compared to the analysis in Figure 1B. (C) Response of left and right dACC. Highlighted regions show where activity is significantly different between sides. There are no changes compared to the analysis in Figure 2A. (D) Averaged responses, coherence and coherence spectrum between right and left ACC during the –200 ms preparatory window. There are no changes compared to the analyses in Figures 4A–C. (E) Pairwise coherence for all electrodes at 24–32 Hz with a 22 ms lag. Each area is represented by a labeled part of the external circle (L on the left, R on the right; the numbers indicate the specific electrodes used on the implanted system). The lines joining sections indicate coherence between the joined regions. The darker the color of the connection the greater the coherence between the joined regions. There are no changes compared to the analysis in Figure 4D. (F) Averaged responses, coherence and coherence spectrum between right and left ACC during the 100–500 ms feedback window. There are no changes compared to the analyses in Figures 5A–C. (G) Pairwise coherence for all electrodes at 8–16 Hz with a 15 ms lag. There are no changes compared to the analysis in Figure 5D. [file Image_6.JPEG]
